# A descriptive longitudinal study protocol: recurrence and pregnancy post-repair of obstetric fistula in Guinea

**DOI:** 10.1186/s12884-016-1101-y

**Published:** 2016-10-10

**Authors:** Alexandre Delamou, Therese Delvaux, Abdoul Habib Beavogui, Alain Levêque, Wei-Hong Zhang, Vincent De Brouwere

**Affiliations:** 1 Ecole de Santé Publique, Université libre de Bruxelles (ULB), Brussels, Belgium; 2 Centre national de formation et de recherche en santé rurale de Maferinyah, Forecariah, Guinea; 3 Department of Public Health, Institute of Tropical Medicine, Antwerp, Belgium; 4 Woman and Child Health Research Centre, Institute of Tropical Medicine, Antwerp, Belgium

**Keywords:** Recurrence, Pregnancy, Risk factors, Obstetric fistula, Post-repair, Guinea

## Abstract

**Background:**

Obstetric fistula is a serious medical condition which affects women in low income countries. Despite the progress of research on fistula, there is little data on long term follow-up after surgical repair. The objective of this study is to analyse the factors associated with the recurrence of fistula and the outcomes of pregnancy following fistula repair in Guinea.

**Methods:**

A descriptive longitudinal study design will be used. The study will include women who underwent fistula repair between 2012 and 2015 at 3 fistula repair sites supported by the Fistula Care Project in Guinea (Kissidougou Prefectoral Hospital, Labé Regional Hospital and Jean Paul II Hospital of Conakry).

Participants giving an informed consent after a home visit by the Fistula Counsellors will be interviewed for enrolment at least 3 months after hospital discharge The study enrolment period is January 1, 2012 − June 30, 2015. Participants will be followed-up until June 30, 2016 for a maximum follow up period of 48 months. The sample size is estimated at 364 women.

The cumulative incidence rates of fistula recurrence and pregnancy post-repair will be calculated using Kaplan-Meier methods and the risk factor analyses will be performed using adjusted Cox regression. The outcomes of pregnancy will be analysed using proportions, the Pearson’s Chi Square (*χ*2) and a logistic regression with associations reported as risk ratios with 95 % confidence intervals. All analyses will be done using STATA version 13 (STATA Corporation, College Station, TX, USA) with a level of significance set at *P* < 0.05.

**Discussion:**

This study will contribute to improving the prevention and management of obstetric fistula within the community and support advocacy efforts for the social reintegration of fistula patients into their communities. It will also guide policy makers and strategic planning for fistula programs.

**Trial registration:**

ClinicalTrials.gov Identifier: NCT02686957. Registered 12 February 2016 (Retrospectively registered).

## Background

Obstetric fistula (OF) is a serious medical condition in which a perforation develops between vagina, bladder and/or rectum, most commonly after prolonged labour when the head of the unborn child compresses the birth canal and leads to tissue necrosis [1, 2]. A recent study in 5 countries showed that women suffering from OF are generally married young, have little formal education and for most of them the fistula occurred after the first pregnancy, at a median age of 20 years [3]. The condition hardly exists in the developed world, but remains prevalent in sub-Saharan Africa, especially in countries where access to quality emergency obstetric care is low [4, 5]. Guinea for instance has a high maternal mortality ratio (724 per 100 000 live births) and a prevalence of OF among women of reproductive age estimated to be 0.6 %, which represents a total number of 15 − 18 thousands women living with an OF [6].

Thanks to the international mobilization against fistula in recent years, the holistic care of OF (prevention, treatment and reintegration) in many sub-Saharan African countries has improved [7, 8]. As a result, immediate post-operative closure rates of OF have increased [9–12]. In addition, the number of repaired women has increased worldwide with more than 30,000 fistula repairs supported by the Fistula Care Project in 15 countries since 2005 [13] and more than 57,000 repairs funded by the UNFPA over the past decade [14]. Furthermore, new evidence has shown that women’s hospital stay can be shorten and costs lowered, allowing more women to be treated [15]. In Guinea, about 3,000 fistulas were repaired between 2007 and 2013 by the Fistula Care Project (a USAID-funded project managed by EngenderHealth) [13, 16].

Despite these improvements, gaps still exist, especially in the long term follow-up of women after successful fistula repair [17]. In fact, data currently available show that delivery leading to fistula usually ends up with a stillbirth [10–12] and that women are relatively young at the time their fistula is repaired [3]. Therefore, when successfully repaired, women return to their community with the desire to resume social and sexual life, and have children [18–20]. However, this return to sexuality exposes repaired woman to pregnancy and childbirth, which increases the risk of recurrence of a previously repaired fistula or the formation of a second, new fistula [18, 19]. Fear of recurrence of fistula is a constant worry for women successfully repaired of OF [21–23] and, given the cost of fistula care and the lower success of repairing [24], these women might need more attention to prevent recurrence of fistula and adverse pregnancy outcomes.

There is no precise estimate of the recurrence of fistula or pregnancy after OF repair. In Ethiopia, Nielsen et al [18] observed 2.63 % re-occurrence of fistula among 38 women in a 21 months’ post-repair median follow up and Browning and Menber [19] recorded 4.26 % in 141 women successfully repaired after 6 months’ follow-up. In Malawi, Wilson et al [20] reported 3.85 % of re-occurrence in 26 women after 9 − 24 months’ follow-up post-repair.

The same authors found a proportion of pregnancies of respectively 13.16 % [18] and 4.26 % [19] in Ethiopia and 23.10 % in Malawi [20]. Delivery outcomes were poor with only 1 live birth reported in both Ethiopia and Malawi [18, 20]. Heavy work, sexual intercourse, and delivery have been reported to be associated with the recurrence of fistula [25–28]. Because sexuality and desire of children are part of women’s life after successful OF repair [18, 19, 21, 29] and recurrence of fistula and stillbirth are very common complications of delivery post OF repair [18, 20], there is a need for more well powered long term follow-up studies that could fill the existing gap of knowledge. The need to collect long-term follow-up data about fistula recurrence, subsequent pregnancies and their outcomes has already been identified among current research priorities on fistula [17]. Since, there has been no major comprehensive and well powered study initiated in a sub-Saharan African country involving women after OF repair, and for the status of the fistula at discharge is not always specified in the existing studies, we initiated this study with the following objectives:

- Analyse the incidence rate and risk factors of fistula recurrence up to 48 months follow up.
- Analyse the incidence rate and factors associated with pregnancy and pregnancy outcomes (maternal and perinatal) up to 48 months’ follow-up;

The endpoints will be recurrence of fistula, pregnancy, abortion or miscarriage, maternal, perinatal and child death at any time from hospital discharge.

## Methods

### Study design

**Fig. 1:**
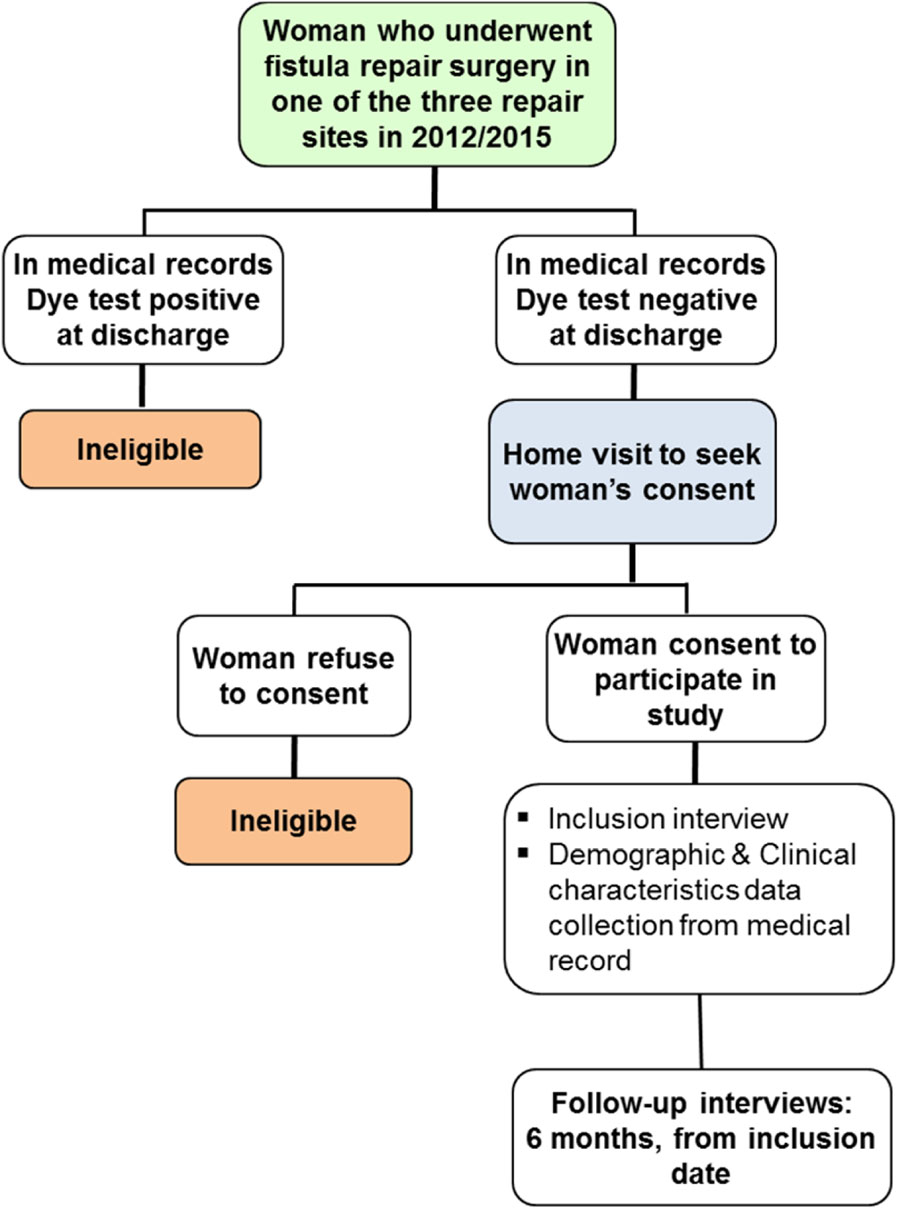
Study flow chart

This is a longitudinal follow-up study over a period of 4 years conducted among women underwent fistula repair at 3 sites supported by Engenderhealth in Guinea. A cohort will be constituted. Participants who have been repaired at OF treatment sites before the study has started will be included retrospectively and then followed up prospectively (Fig. 1).

The primary outcome is the incidence rate of the recurrence of fistula. For the purpose of this study, recurrence of fistula includes the breakdown of a repaired fistula or the occurrence of a new fistula in study participants. At the study sites, the closure of fistula was determined at hospital discharge by a pelvic exam using a dye test. During follow-up visits, women will first be asked about current medical status using the question “does the woman have continuous and uncontrolled leakage of urine?”. If the answer is “YES” then women will be scheduled for a pelvic exam with a dye test for confirmation, at the nearest health facility (health centre or health post). This question is always used at EngenderHealth supported sites to assess women at reception, before further pelvic exam. In addition, the presence of “continuous and uncontrolled leakage of urine” is commonly used as a screening question for fistula in circumstances in which a pelvic exam is not possible (e.g. Demographic and Helath Surveys, DHS); it differentiates between fistula and other forms of incontinence, which are unlikely to be continuous and uncontrolled. In some cases, it might not be feasible to perform the dye test if for instance the woman refuses to go to a health facility. This might lead to a misclassification which can bias the estimate towards a null. However, misclassification is unlikely, since women with continued urinary leakage are eager to report their experiences in order for the condition to be rectified.

The secondary outcomes include pregnancy and pregnancy and outcomes (miscarriage or abortion or term pregnancy). Pregnancy and pregnancy outcomes will be documented by a positive pregnancy test from discharge (done at recruitment or during follow-up visit or given at any time by a result from medical records such as antenatal (ANC) card) or by a self-reporting corroborated by 2 witnesses. Maternal status at delivery and mode of delivery (dead or alive; caesarean section or normal delivery; no complication or any complication) will be assessed by looking at the ANC & Delivery card or hospital records or self-reporting. Child outcome at delivery (stillbirth, neonatal death or alive) will be assessed by looking at the ANC & Delivery card or hospital records or self-reporting corroborated by 2 witnesses.

Our predictors of interest will include patient characteristics, fistula characteristics, context of repair and context of reintegration. Because women will be recruited and followed-up to record the occurrence of the study outcomes over a period of time, the longitudinal follow-up study design is the most suitable design.

### Study setting

#### General setting

Guinea is a coastal West African country with an estimated population of 12 million people [6]. The population is largely rural (65 %) and poor [30]. The Guinean health care system faces many challenges, including chronic shortage of qualified physicians and nurses, and poor clinical infrastructure, particularly in rural and mountainous areas. According to the national action plan for repositioning family planning in Guinea 2014–2018 [20], only 16 % of health professionals (doctors, state midwives and state nurses) work in rural areas where they serve 65 % of the population. Guinea has an estimated lifetime prevalence of obstetric fistula of 0.6 % among women aged 15–49 which ranges regionally from 0.2 to 1.2 % [6], although this figure is likely underestimated. Challenging contextual factors in maternal health include low modern contraceptive prevalence (6 %), concurrent high fertility rate of 5.1, and high maternal mortality (724 deaths per 100,000 live births) [6]. Over the period 2007–2012, the majority of births (54.7 %) were assisted by untrained individuals, and occurred at home (58.8 %) [6]. Since 2012, the Ministry of Health has elaborated a national strategic plan (2012–2016) for the prevention and treatment of fistula [16]. This strategic plan emphasizes the importance of reintegration and support to victims of OF and advocates for more operational and action research that can inform policy.

#### Specific setting

Fistula repair services are available in 4 sites across the country but the study will be conducted with women successfully repaired at the 3 Engenderhealth supported hospitals. From 2007 to 2013, about 3000 OF repairs have been conducted at the 3 Engenderhealth supported sites (Jean Paul II Hospital in Conakry, the Regional Hospital of Labé and the Prefectural Hospital of Kissidougou), where 16 physicians have been trained in obstetric fistula repair, 55 nurses/midwives have been trained in fistula management and 400 − 450 fistula repairs per year have been performed [13].

### Study population

The study population includes all women who underwent successful repair of OF from January 1, 2012 − June 30, 2015 at the 3 above described fistula repair sites.

### Sample size calculation

For the estimation of the incidence rate of the recurrence of fistula, we used the 2-Sided Confidence Intervals for 1 Proportion Confidence Interval Formula [31]. Using the existing data [19] and considering a +/- 2 % margin of error and 95 % confidence interval, we estimate the sample size of the cohort to be 280. We anticipate 20 % loss to follow-up and 10 % refusal rate and accordingly increase the sample size to 364. The same formula provides an estimation of the sample size for the incidence rate of pregnancy [31]. If we estimate the rate of pregnancy to be around 20 % at 24 months’ follow-up among successfully repaired women [20], a sample size of 264 women produces a 2-sided 95 % confidence interval with 10 % precision. Therefore, the sample size of 364 women will be sufficient to describe the objective on pregnancy and its outcomes.

### Participants’ recruitment and follow-up

All women who underwent successful repair of OF from January 1, 2012 to June 30, 2015 at the 3 study sites are eligible for inclusion in study. However, only women with a unique obstetric fistula that was closed at discharge, as measured by a dye test, who provide written informed consent, reside in Guinea and agree with follow-up visits will be included in the study (Fig. 1). Eligible participants who refuse informed consent, who got repair outside the study sites or whose medical records are incomplete will be excluded.

Individual medical records of all women who benefited fistula repair at the 3 sites in 2012–2015 will be screened by the study team to identify those who were discharged with a close fistula (Fig. 1). After locating them through their medical records, the study team (that includes nurses called “Fistula Counsellors” who are involved in the management of women at the fistula repair sites) will contact women by home visit in their community to obtain informed consent.

Once the informed consent is obtained, the woman will be interviewed for 30 − 60 min by the study team and examined in an isolated place for inclusion in the study. Later, her medical records will be used at the fistula repair site where she got the surgery to collect her sociodemographic and clinical characteristics at the time of surgery. For all women recruited in the study, most retrospectively, new fistula or recurrence of the repaired fistula, pregnancies and their outcomes (miscarriage, stillbirth, live birth, early new-born death, maternal death, and type of maternal complication) will be documented. Health Centres and Health posts around which the study participants live will be used by the study team to perform the dye test in women who report “Continuous and uncontrolled leaking” since discharge or at the follow up visit.

### Study procedures

**Fig. 2:**
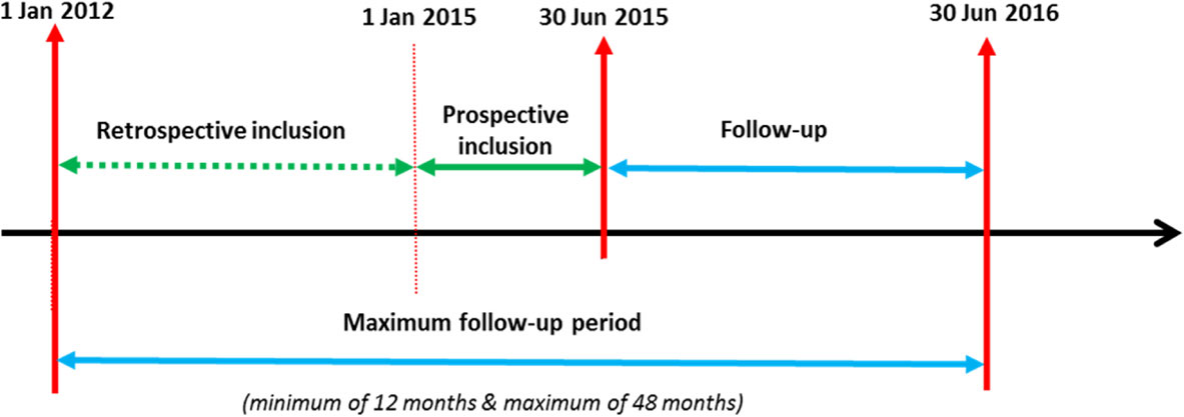
Study timeline

The study team will retrospectively screen individual medical records of all women who benefited fistula repair at the 3 sites from January 1, 2012 to June 30, 2015 to identify those who were discharged with a close fistula (Table 1 and Fig. 2).

**Table 1:** Study schedule of assessment

Study steps	Procedures
*Screening*	• Retrieving of medical records • Identification of women closed at discharge
*Enrolment*	• Home visit to seek informed consent • Administering of inclusion questionnaire • Dye test at nearest health post/centre • Appropriate referral when needed • Collection of demographic and clinical data from medical records
*Follow up visits*	• Home visit to administer follow up questionnaire • Dye test at nearest health post/centre • Appropriate referral when needed

A home visit will then be conducted within the community to seek informed consent with the repaired women. After obtaining the consent, women will be interviewed. Women who report “Continuous and uncontrolled leaking” since the last follow-up visit will be scheduled for a pelvic exam and a dye test at the nearest health centre or health post. If the case is confirmed as fistula (by a dye test of clinical exam), the woman will be counselled and referred to the nearest fistula repair centre. By this approach, we think that any misclassification of outcome (recurrence of fistula) will be minimal, since women with any continuous and uncontrolled leakage of urine following repair are likely to report it, and any reported incontinence will be evaluated by a dye test.

If a woman reports pregnancy, she will be asked to show her antenatal care (ANC) & delivery card on which pregnancy test result is routinely reported. If possible, a pregnancy test will be performed. Otherwise, investigators will rely on woman self-reporting.

Sociodemographic and clinical characteristics of women will be collected from individual medical records at each participating fistula repair site.

All investigators will receive appropriate training at the Maferinyah National Research and Training Centre before they go in the field.

### Data collection

#### Recruitment and follow-up visit

The enrolment and follow up data are collected using structured and pre tested standardised questionnaires. Both questionnaires will include participants’ current fistula status (fistula close and continent/closed but not continent), post-operative social and reproductive life (marital status, occupation, sexuality, use of modern contraceptive methods, pregnancy, childbirth), and any fistula related complication. Participants’ current full address is also collected to facilitate communication, referral and follow-up visits.

#### Sociodemographic and clinical characteristics

After obtaining the informed consent during the recruitment visit, a standardised questionnaire called Baseline form is used to collect the demographic and clinical characteristics of women from hospital records in the hospitals where they were discharged from. This questionnaire includes age at presentation at hospital, marital status, age at marriage, occupation, level of education, residence (rural/urban), number of pregnancies, parity, number of previous repairs, and duration of fistula. Clinical characteristics include mode of delivery related to development of the fistula, neonatal outcome at causal delivery, type of fistula (VVF, RVF or both), status of the bladder neck, nature of the fistula according to the surgeon (simple versus complex), duration of catheterization after repair, status of the operating surgeon (expatriate, local trainer, local trainee), postoperative complications, context of repair (routine repair or pooled repair) and the status of the fistula at discharge (close and continent/Close but not continent).

### Data management

Study forms will be checked after data collection before sending to the Maferinyah Training and Research Centre in a sealed envelope where they will be stored in a locked cabinet. Data will be double entered by 2 independent encoders into a data entry file created using EpiData Entry software (EpiData Association, Odense, Denmark). Reliability will be checked and any inconsistencies in data entry will be resolved. It is planned to perform data entry on a continuous basis from the start of enrolment so that discrepancies are addressed while data collection is ongoing. Data entry screens will be created for each form used in the study. The database will be password protected and only authorized users will be given access. All data will be stored in a GCP compliant server, and data management will respect patient confidentiality.

All study documents with the participant’s name (i.e. participants’ contact form) will be kept in a strict confidentiality.

### Data analysis

Categorical data and treatment outcomes will be summarized as frequencies (%) and compared using *χ*2 or Fisher’s exact tests as appropriate. Continuous data will be presented using median (interquartile range) and compared using the Wilcoxon rank-sum test. Follow-up will be calculated from the date of hospital discharge. The date of fistula recurrence or the estimated date of pregnancy will be selected as the date of events. Patients who do not experience recurrence of fistula or pregnancy will be censored at the last follow up visit. For patients who are reported dead (by the relatives), the date of death will be used to determine their follow-up time in the study. For patients who are lost to follow up (i.e. who move from their village without any address to joint them after inclusion) the date of the last visit will be used.

Socio-demographic and clinical differences between those women who remain in the study and those who are lost-to-follow-up will be estimated to check for any imbalances at inclusion.

The incidence of fistula recurrence and that of pregnancy will be estimated using the method of cumulative incidence. The cumulative incidence rate will be compared across risk factors using Kaplan-Meier methods. Exposure variables with a *P*-value <0.2 in the univariate analysis will be included in multivariate models. Exposure variables with a *P*-value <0.2 in the univariate analysis will be included in multivariate models. A risk factor analysis will be performed using adjusted Cox regression. Associations will be reported as adjusted hazard ratios with 95 % CIs. Analysis will be done using STATA version 12 (STATA Corporation, College Station, TX, USA) with a level of significance will be set at *P* < 0.05.

For the outcomes of pregnancy (miscarriage or abortion, mother and child status at delivery), Pearson’s Chi square (*χ*2) will be used to compare proportions of these outcomes between potential exposure variables (age group, previous repair (Yes/No), fistula status at hospital discharge, use of antenatal care, delivery by caesarean section, etc.). Logistic regression analysis and models will be used and associations will be reported as risk ratios with a level of significance set at *P* = 0.05 and a 95 % confidence intervals.

## Discussion

This longitudinal study will generate long-term follow-up data on fistula recurrence, subsequent pregnancies and their outcomes among women who underwent successful fistula repair in order to guide policy making and strategic planning in Guinea. This will improve current and future programmes for fistula management and care. The findings will also contribute towards improving programme performance and support advocacy efforts for better reintegration of fistula patients in Guinea and beyond.

This study has a number of strengths. First the protocol employs appropriate design and analytical methods, and a well powered sample size. Second, it will take advantage of the large collection of long term data assessing the recurrence of fistula and the outcomes of pregnancy. Third, it will add to previous studies examining fistula recurrence and reproductive outcomes after obstetric fistula repair by assessing a wide variety of variables, and by adjusting for potential confounding factors.

The results of this study and its potential implications will be made known to health care staff in the study sites, as well as the national level. National and international platforms will be used to disseminate study findings and the results will be presented at international conferences and published in peer reviewed journals. Since the findings might be applicable to other fistula care and management programmes in neighbouring countries, the lessons learnt might have wider benefit.

## Abbreviations

ANC: Antenatal care
CI: Confidence intervals
DHS: Demographic and Health Survey
GCP: Good Clinical Practice
IRB: Institutional Review Board
ITM: Institute of Tropical Medicine
OF: Obstetric fistula
RVF: Rectovaginal fistula
UNFPA: United Nations Population Fund
USA: United States of America
USAID: United States Agency for International Development
VVF: Vesicovaginal fistula
